# The anomalous effect of electric field on friction for microscale structural superlubric graphite/Au contact

**DOI:** 10.1093/nsr/nwae019

**Published:** 2024-01-25

**Authors:** Yelingyi Wang, Jin Wang, Tielin Wu, Weipeng Chen, Deli Peng, Zhanghui Wu, Ming Ma, Quanshui Zheng

**Affiliations:** Center for Nano and Micro Mechanics, School of Aerospace Engineering, Tsinghua University, Beijing 100084, China; Department of Engineering Mechanics, School of Aerospace Engineering, Tsinghua University, Beijing 100084, China; International School for Advanced Studies (SISSA), Trieste 34136, Italy; International Centre for Theoretical Physics (ICTP), Trieste 34151, Italy; Center for Nano and Micro Mechanics, School of Aerospace Engineering, Tsinghua University, Beijing 100084, China; Department of Engineering Mechanics, School of Aerospace Engineering, Tsinghua University, Beijing 100084, China; Center for Nano and Micro Mechanics, School of Aerospace Engineering, Tsinghua University, Beijing 100084, China; Department of Engineering Mechanics, School of Aerospace Engineering, Tsinghua University, Beijing 100084, China; Department of Engineering Mechanics, School of Aerospace Engineering, Tsinghua University, Beijing 100084, China; Institute of Superlubricity Technology, Research Institute of Tsinghua University in Shenzhen, Shenzhen 518057, China; Department of Engineering Mechanics, School of Aerospace Engineering, Tsinghua University, Beijing 100084, China; Center for Nano and Micro Mechanics, School of Aerospace Engineering, Tsinghua University, Beijing 100084, China; Institute of Superlubricity Technology, Research Institute of Tsinghua University in Shenzhen, Shenzhen 518057, China; Department of Mechanical Engineering, State Key Lab of Tribology in Advanced Equipment (SKLT), Tsinghua University, Beijing 10084, China; Center for Nano and Micro Mechanics, School of Aerospace Engineering, Tsinghua University, Beijing 100084, China; Department of Engineering Mechanics, School of Aerospace Engineering, Tsinghua University, Beijing 100084, China; Institute of Superlubricity Technology, Research Institute of Tsinghua University in Shenzhen, Shenzhen 518057, China; Institute of Materials Research, Tsinghua Shenzhen International Graduate School, Shenzhen 518057, China

**Keywords:** current-carrying friction, structural superlubricity, water, sliding electrical contact

## Abstract

The current-carrying friction characteristics are crucial for the performance of a sliding electrical contact, which plays critical roles in numerous electrical machines and devices. However, these characteristics are influenced by multiple factors such as material surface quality, chemical reactions, and atmospheric environment, leading to a challenge for researchers to comprehensively consider these impacts. Structural superlubricity (SSL), a state of nearly zero friction and no wear between contact solid surfaces, provides an ideal experimental system for these studies. Here, with microscale graphite flakes on atomic-flattened Au surface under applied voltages, we observed two opposite friction phenomena, depending only on whether the edge of graphite flake was in contact with the Au substrate. When in contact the friction force would increase with an increasing voltage, otherwise, the friction force would decrease. Notably, when the voltage was turned off, the friction force quickly recovered to its original level, indicating the absence of wear. Through atmosphere control and molecular dynamics simulations, we revealed the mechanism to be the different roles played by the water molecules confined at the interface or adsorbed near the edges. Our experimental results demonstrate the remarkable tunable and robust frictional properties of SSL under an electrical field, providing an ideal system for the fundamental research of not only sliding electrical contacts, but also novel devices which demand tunable frictions.

## Teaser

This work explores how electric field affects friction in microscale structural superlubric graphite/Au contacts, demonstrating the remarkable tunable and robust frictional properties of structural superlubricity under an electric field.

## INTRODUCTION

Current-carrying friction is a ubiquitous phenomenon observed in sliding electrical contacts (SECs) [1–4]. Extensive studies have revealed that the presence of an electric current intensifies the severity of friction and wear in the contacting components [5,6]. This aggravation is further compounded by various other factors [7–9], including electrical erosion and electrical breakdown, accelerating the failure of SECs. Therefore, it is necessary to understand the reasons for the failure in order to improve the contacts’ electrical and tribological properties.

For traditional SECs which are usually based on metal, their surface roughness is typically between 0.1 μm to 10 μm as limited by current manufacturing techniques [10,11]. With such SECs, and the flow of electric current across a rough interface, due to the necking effects, Joule heating will occur at multiple contact points [12]. As a result, the presence of thermal pressure and localized deformation caused by sliding and thermal pressure easily disrupts the stable lubricating film formed on the friction surface [13–16]. On the one hand, the fragments of these oxides become detached from the surface, serve as abrasive particles, and thereby induce substantial wear [13,14]. On the other hand, the newly exposed metal surface will experience oxidation, leading to a locally rougher surface, which will also increase friction and wear [16]. These complex factors contribute to the intricate nature of the interface environment, making it challenging to analyze and comprehend its state accurately.

Structural superlubricity (SSL) is a state showing nearly zero friction and no wear between two solid contact surfaces [17–19]. The characteristics of SSL interfaces are atomically smooth and in full contact with weak van der Waals interaction across the interface [20–23]. As a graphite flake is the most commonly used SSL material and it holds ultrahigh intra-plane conductivity [24,25], reasonably high inter-plane conductivity [26] and good thermal [27] and mechanical stability [28,29], together with the atomically smooth morphology [20], it is reasonable to assume that the challenges usually met in traditional SECs as mentioned above could be largely solved. To this end, a graphite SSL contact could also serve as an ideal system for studying current-carrying friction. However, up to now, there has been little research on how the friction would be influenced by the electrical current for the SSL state.

In this study, we investigate the friction properties between graphite and Au under different voltages. By increasing the voltage, we find that the friction increases or decreases depending on whether the edge of the graphite flake is in contact with the Au substrate or not. In both cases, the low friction and zero wear characteristics of the contacts are maintained, showing SSL. With comparative experiments and molecular simulation, we show that the intricate roles played by the adsorbed water molecules is the key to understanding the mechanism.

## RESULTS

**Figure 1.**
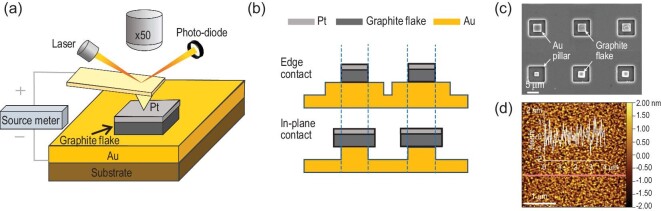
Experimental set-up and samples for current-carrying friction measurements. (a) Experiment set-up. (b) Two contact states of graphite and Au: the ‘edge contact’ (top part) and ‘in-plane contact’ (sub part). (c) The SEM image of the two types of contacts, corresponding to the states illustrated in (b). (d) The topography of the Au surface by AFM scanning. The inset showing the profile of the red line.

Our samples were fabricated by lithography on highly oriented pyrolytic graphite (HOPG) with a 200 nm thick Pt film (fabrication details are provided in Methods). We chose a type of graphite flake which shows self-retraction motion, i.e. the top part of the flake will return to its initial position after being pushed away. This spontaneous return motion suggests the SSL property of the flake [30,31]. We transferred the graphite flake with self-retracting motion to Au substrates by shearing the top of the graphite flake (transfer details are provided in Supplementary Section 1). The Au film, with a thickness of 100 nm, was deposited on the silicon (1 1 1) surface by physical vapor deposition. The experiment was conducted using a home-built source meter-atomic force microscope (AFM) platform, as illustrated in Fig. 1a. A visible AFM tip (VIT_P AFM probe, NT-MDT, Russia) was in close contact with the Pt cap to restrict the motion of the graphite flake. Therefore, a horizontal displacement controlled by the scanner could cause a shear motion between graphite flake and Au substrate. For each friction measurement detected by an AFM tip, the force was calibrated *in situ* just after the friction measurement, using the Sader method [32] for the normal direction and the diamagnetic lateral force calibration method [33] for the lateral direction. By connecting an external source meter (Keithley 2450) to the AFM, a constant electrical voltage between 0 and 5 V was applied between the AFM tip and Au substrate to provide a large measuring scale.

Figure 1b shows two different contact states between the graphite flake and Au substrate in our experiments: one where the edge of the graphite flake was in contact with the substrate's surface (noted as ‘edge contact’, upper part of Fig. 1b), and the other where the edge of the graphite flake was suspended with in-plane contact (noted as ‘in-plane contact’, lower part of Figure 1b). The process of fabricating Au pillars is shown in Supplementary Section 2. Fig. 1c displays pictures of the two contact states, photographed by a scanning electron microscope (SEM). During the friction tests, we maintained the edge of the graphite flake in-contact and out-of-contact with the top surface of the Au pillar for ‘edge contact’ and ‘in-plane contact’, respectively. The Au pillars with a height of 1 μm as shown in Fig. 1c was made from Au substrate using lithography. A typical morphology of the top surface for the Au pillar characterized by AFM is shown in Fig. 1d, with a roughness of Ra = 0.6 nm. According to the formular $H > {H}_{\mathrm{c}} = 3Ea{h}^2/16( {\gamma a + {p}_{{\mathrm{zz}}}ha} )$ provided by Peng *et al.* [29], our graphite flake could achieve full contact with the substrate (details in Supplementary Section 3). As a result, the real contact area can be considered equal to the apparent contact area. We transferred 4 μm graphite flake onto 6 μm pillar, and 6 μm graphite flake onto 4 μm pillar, respectively, to keep the same contact area (*A*) for the two states, both 16 μm^2^.

**Figure 2.**
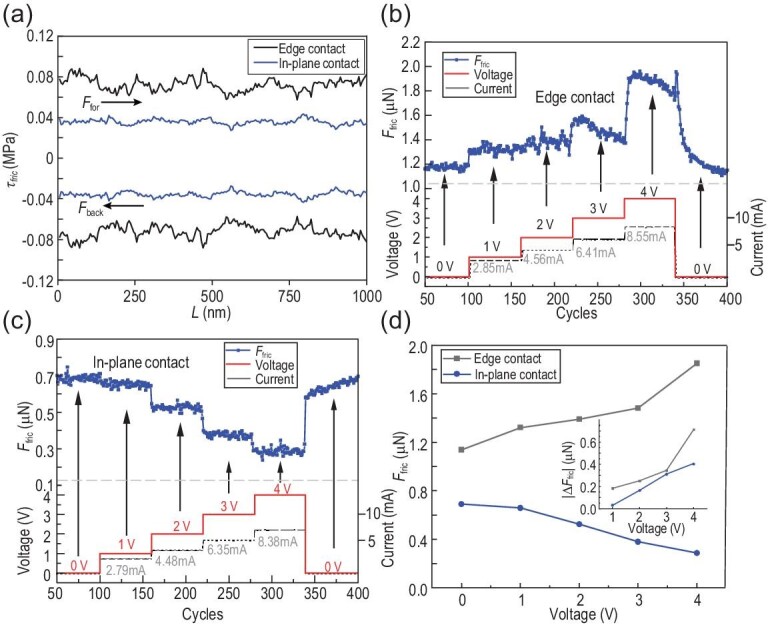
The opposite changing trends of the two types of contact states. (a) The frictional stress loop between graphite and Au, black for ‘edge contact’ and blue for ‘in-plane contact’. (b and c) The variation of friction caused by the applied voltages for ‘edge contact’ and ‘in-plane contact’, respectively. We increased the applied voltages from 0 V to 4 V each by 1 V and kept every voltage for ∼70 sliding cycles, and at last, returned the voltages to 0 V. (d) The changes of friction at each voltage for both contact states. The inset shows the relationship between $\Delta {F}_{{\mathrm{fric}}}$ and voltages, where ${\mathrm{\Delta }}{F}_{{\mathrm{fric}}} = {F}_{{\mathrm{vol}}} - {F}_0$, and ${F}_{{\mathrm{vol}}}$ is the friction force at each voltage,${\mathrm{\ }}{F}_0$ is the friction force without voltage.

Before measurements, we slid the graphite flake back and forth for more than 500 cycles under 110°C to remove some interface contaminants [34,35]. The following experiments were conducted under ambient conditions (temperature: ∼25°C, relative humidity (RH): 40%–50%) unless otherwise specified. Figure 2a illustrates the friction loop between the graphite flake and Au (black for ‘edge contact’ and blue for ‘in-plane contact’), directly obtained from the signal of lateral force detected by AFM systems during the measuring process. (See online supplementary material for a color version of this figure.) The area enclosed by the loop denotes the energy dissipation caused by friction, that is ${U}_{{\mathrm{dis}}} = ( {{F}\,_{{\mathrm{for}}} - {F}\,_{{\mathrm{back}}}} )L = 2L{F}\,_{{\mathrm{fric}}}$ where ${U}_{{\mathrm{dis}}}$ is the energy dissipation, ${F}_{{\mathrm{for}}}$ and ${F}_{{\mathrm{back}}}$ are the average lateral forces of forward and backward sliding, *L* is the sliding scale and thus $2L$ is the sliding distance of a single friction loop. Therefore, the friction force ${F}_{{\mathrm{fric}}}$ and frictional stress ${\tau }_{{\mathrm{fric}}}$ can be calculated as ${F}_{{\mathrm{fric}}} = | {{F}_{{\mathrm{for}}} - {F}_{{\mathrm{back}}}} |/2$ and ${\tau }_{{\mathrm{fric}}} = {F}_{{\mathrm{fric}}}/A$, respectively. Consequently, the average frictional stress is <0.04 MPa for ‘in-plane contact’ and ∼0.08 MPa for ‘edge contact’, at 50 μN normal load which amounts to 3.1 MPa. Such an extremely low friction is a typical feature of SSL [36]. The ‘edge contact’ frictional stress is approximately 2 times larger than that for ‘in-plane contact’, which might be caused by the energy dissipation from the out-of-plane motion of the edge atoms of the slider [37,38].

Next, we measured the friction force continuously while applying voltages between graphite flake and Au substrates to observe the variation of friction both for edge contact and in-plane contact. We increased the voltages from 0 to 4 V with a step of 1 V then decreased back to 0 V. As shown in Fig. 2b and c, interestingly, the changing trends for the two cases were totally opposite depending on the contact states. Specifically, the friction would increase and decrease with the increasing of voltages for ‘edge contact’ and ‘in-plane contact’, respectively. It is worth mentioning that the friction would be recovered quickly to the original level for both contacts, indicating the absence of permanent changes occurring during the friction process under different voltages. What's more, the friction responds quickly to the increase in voltage, within 5 cycles, demonstrating great potential in applications requiring friction tuning. Furthermore, we conducted friction experiments under cyclic voltages, and the experimental results are shown in Supplementary Section 4, indicating a well-established reproducibility in the relationship between interfacial friction and voltage. In addition, we present the relationship between friction and applied voltages in Fig. 2d, and the inset shows the magnitude of friction variation at different voltages. Applying an electric field for ‘edge contact’ results in a gradual increase in friction force within the range of 0–3 V. At 4 V, there is a notable elevation of ∼0.7 μN, representing a substantial 50% increase compared to the initial friction force. Conversely, for in-plane contact with the application of an electric field, the friction force exhibits a smoother decline, reaching 0.25 μN at 4 V, equivalent to 35% of the initial friction force.

**Figure 3.**
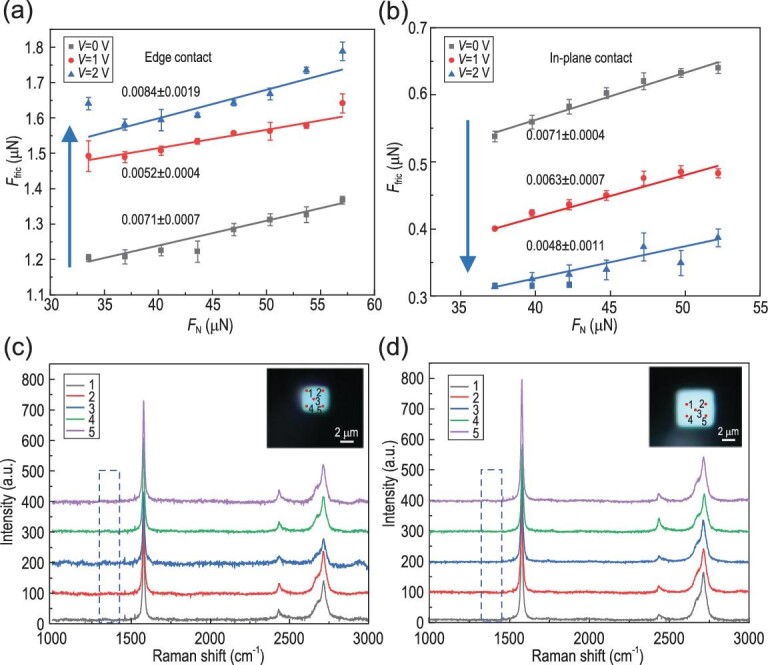
SSL characterization of both contacts. (a and b) The relationship between friction ${F}_{{\mathrm{fri}}}$ and normal load ${F}_{\mathrm{N}}$ for ‘edge contact’ (a) and ‘in-plane contact’ (b), and obtaining the coefficients of friction (COFs) by linear fitting using least squares. (c and d) Raman spectra of the sliding interfaces of graphite flake for ‘edge contact’ (c) and ‘in-plane contact’ (d). The insets of (c and d) are the optical images of graphite flakes’ sliding interfaces, and the red points represented the characterized positions, corresponding to the lines in (c and d).

Besides friction, we further measured the effects of voltage on the differential coefficient of friction. Figure 3a and b shows the relationship between friction force and normal load of the two contacts, respectively. We obtained the differential coefficients of friction (dCOFs) for each group of experiments by linear fitting, and the results showed that dCOFs were all in the order of 0.001 and nearly remained the same. Furthermore, after the friction test, we characterized the defect information of the sliding interfaces of graphite flakes by Raman spectroscopy. Using a tungsten tip with a glue drop to pick up and flip the graphite flake, we can intuitively study the lower surface of the graphite (preparation details are provided in Supplementary Section 5). As shown in Fig. 3c and d, the absence of D peak shows that no defect occurred in both contacts, meaning that there was no wear or damage, which agrees with the reversible friction observed while changing the voltage. Together with the low friction stress (on the order of 10 kPa), the graphite flakes were in SSL state with Au substrates during the whole experiment [29,39,40].

For graphite homojunction, Wang *et al.* proved that water molecules on graphite contacts could not be eliminated and the structural superlubric feature is well preserved [41]. In our experimental system where graphite-Au heterojunctions were studied, Au film was fabricated on silicon (111) substrate through physical vapor deposition with an arithmetic mean roughness (Ra) of 0.6 nm which is rougher than graphite surface. In this case, we suspected that we can hardly eliminate the interlayer water between graphite and Au. Previous research has shown that water molecules in the electric field have a variety of effects on friction with completely different mechanisms [42–44]. For instance, water molecules could develop into ice-like water layers between the AFM tip and graphene to increase friction [42]. Inspired by such observations, we suppose that the interfacial water molecules between graphite flake and Au could account for the intriguing phenomena observed.

Therefore, we repeated the experiments under nitrogen environment with different humidity to examine the effects of confined water. The experiments were carried out in a home-built chamber full of nitrogen gas. We transferred our graphite flake to the Au substrates at a temperature of 150°C under nitrogen environments to avoid the introduction of water molecules. After naturally cooling down to ambient temperature, we performed the friction test with nitrogen atmosphere, serving as a reference system. Afterwards, for comparison, we controlled the nitrogen's RH to ∼40% to match our previous experimental conditions by passing the nitrogen through water using a gas washer bottle.

**Figure 4.**
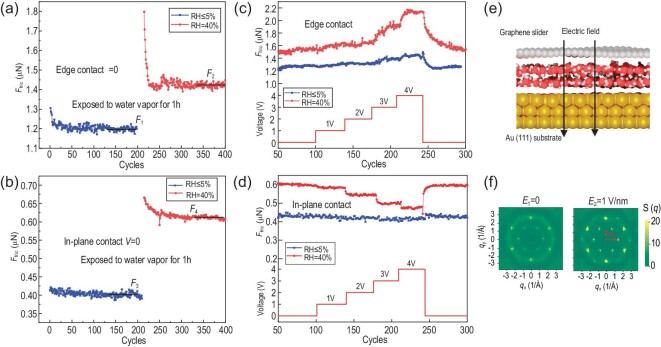
The mechanism of the variation of friction in both contacts. All experiments in this section were conducted under nitrogen environment with different concentrations of water vapor. (a and b) The comparison of friction forces under low humidity (5%) and high humidity (40%) without electrics for ‘edge contact’ and ‘in-plane contact’, respectively. (c and d) The variation of friction forces with different applied voltages under low humidity (5%) and high humidity (40%) for the two types of contacts. (e) The illustration of MD model. (f) The structure factor of the oxygen atoms at the interface for E = 0 and 1 V/nm.

First, we measured the friction between graphite and Au with ‘edge contact’ under dry nitrogen circumstances without voltage, and the friction would be stable after several cycles, noted as ${F}_1$, as Fig. 4a shows. Then, water was introduced to increase the RH to 40%. After 1-h maintenance, we measured the friction again and found that it would stay around ${F}_2$ after dozens of cycles. Similarly, Fig. 4b shows the friction between graphite and Au with ‘in-plane contact’. In both cases, we obtained obvious ‘running-in’ processes and larger steady friction after the introduction of water (${F}_2 > {F}_1,\ {F}_4 > {F}_3$), indicating that water inclusions appeared at the sliding interfaces and cannot be eliminated completely.

Then, we applied a voltage from 0 to 4 V between graphite flake and Au substates and measured the friction simultaneously. The blue and red lines in Fig. 4c show the variation of friction depending on voltages of ‘edge contact’ under dry and humid nitrogen conditions, respectively. Interestingly, for dry nitrogen condition (RH ≤5%), the friction force rarely changed with the increasing voltages except for the friction at 4 V. However, after exposure to humid nitrogen, the friction force of the same sample significantly increased with voltages, similar to the experimental phenomenon in Fig. 2b. As for the ‘in-plane contact’, shown in Fig. 4d, it was much more obvious to prove the contribution of water inclusions on the variation of friction tuning by voltages. The friction was almost independent with the applied voltages under dry nitrogen while showing high dependence with voltages under humid conditions, just like that in Fig. 2c. These phenomena proved that water inclusions played an important role in the friction tuning effects by voltages in both types of contacts. Furthermore, we also measured the resistance variation of the sample in both dry nitrogen and humid nitrogen environments, as detailed in Supplementary Section 6. It was observed that when the sample was exposed to humid nitrogen, the system's resistance increased significantly and exhibited instability. This observation also suggests the introduction of water molecules at the graphite/Au interface, which subsequently affects the contact state.

To gain atomic scale understandings on how water inclusions affect the friction under electric fields, simulations of molecular dynamics (MDs) were performed. The simulation model (Fig. 4e) consists of graphene slider, Au (111) substrate, and the intercalated water layer. Considering the huge gap between the largest experimental velocity ($\mu{\mathrm{ m}}/{\mathrm{s}}$) and the typical MD simulations velocity ($\sim {\mathrm{m}}/{\mathrm{s}}$), instead of performing non-equilibrium simulations to estimate the friction (at a crazily high velocity), we here adopted equilibrium simulation protocols to quantify the drift mobility $\mu $ of the graphene flake. The friction of the slider is inversely proportional to its mobility [45], $F \propto {\mu }^{ - 1}$. At low velocity limits, mobility is connected to the diffusion coefficient by Einstein’s relation [46], $\mu = D/{k}_{\mathrm{B}}T$, where *D* is the diffusion coefficient. Therefore, the friction of the system can be qualitatively described by *D*^−1^. Detailed simulation protocols can be found in Methods.

The diffusion coefficient *D* of the graphene slider was calculated for two representative systems, with intensities of electric field in z-direction *E*_1_ = 0 and *E*_2_ = 1 V/nm. Considering that the interfacial distance is on the order of nm, the voltage corresponding to the latter is thus on the order of V, which agrees with the experiments. Note that the diffusion coefficient decreases as the size of the slider increases, this is however, expected from the fact that the friction increases with the contact size. Thus, one should focus on the relative value of *D* of these two systems rather than on their absolute values. Averaging over three independent simulations, the diffusion coefficient of the two systems is ${D}_1 = 1.33\ \pm \ 0.23\ {\mathring{\rm A}}^2/{\mathrm{ps}}$, ${D}_2 = 1.56\ \pm \ 0.15\ {\mathring{\rm A}}^2/{\mathrm{ps}}$. Since $F \propto {D}^{ - 1}$, the values of *D* indicate that the friction with applied voltages was smaller than those without, agreeing well with our experimental measurements (Figs 2c and 4d). By further analyzing the structure factor of the water layer, we found that the water layer under a finite electric field formed a more distinct crystal-like structure (Fig. 4f). This structured water layer contacted with graphene interfaces incommensurately, and thus reduced the friction [47]. Furthermore, we measured the friction variation of the sample by changing the direction of the electrical field, and performed additional simulations to estimate the friction with parameter *E* = −1 V/nm (details in Supplementary Section 7). The result indicates that the friction-weakening behavior depends weakly on the direction of the electrical field.

Given the periodic boundary conditions employed in the simulations, the model utilized in this study effectively represents the ‘in-plane contact’ observed in the corresponding experimental setup. In contrast, under circumstances characterized by ‘edge contact’, the contribution to friction originating from the edges surpassed that originating from the interior parts. This is validated by the experimental measurements where the friction for edge contact is always significantly larger than that of the in-plane contact (Fig. 2b vs 2c, or Fig. 3a vs 3b, or Fig. 4a vs 4b). In-depth analysis of the infrared spectra and molecular simulations revealed that a substantial portion of the water molecules become adsorbed at the graphite edges, primarily attributable to the presence of dangling bonds and amorphous carbon [48,49]. As the voltage was applied, an increased amount of adsorbed water molecules accumulated at the edge of the graphite flake as a result of the polar nature of water molecules [50–52]. This progressive accumulation of water molecules results in pinning sites across the interface, consequently leading to an additional friction dissipation motion (Figs 2b and 4c).

## CONCLUSIONS

To conclude, our results demonstrate that the graphite/Au interface in the electric field displays a robust SSL state under ambient conditions owing to the resistance to oxidation and abrasion, which is rarely observed in traditional SECs. Interestingly, the friction was tuned by the applied voltages and increased or decreased depending on whether the edge of the graphite flake was in contact with the Au substrate or not. This is beyond the prediction of traditional tribology theories, where for a given contact, the dependence of friction on external electric field is monotonic and definite.

With comparative experiments and MD simulations, we reveal the mechanism to be the different roles played by the confined water molecules at the interfaces and those stuck to the edges. For water molecules confined at the interface, they would arrange in an ordered structure under electrical field, forming an incommensurate interface between graphite and water, thus reducing friction. For those at the edges, they would enhance friction with increasing voltages because of the pinning effect.

Our work showcases the exceptional tunable and robust frictional properties of SSL under an electrical field, emphasizing the potential not only for fundamental research such as the properties of water under extreme confinement with multi-field coupling, but also for sliding electrical SSL contacts in diverse devices that require adjustable friction, such as adjustable torque clutches and adjustable-damping suspension systems in automobiles.

## METHODS

### Preparation of graphite flake

High-quality HOPG (ZYB grade, Brucker [53]) samples of cm size and with a thickness of approximately 2 mm were used as substrates. First, spin on a double layer photoresist LOR 1 A (100 nm)/ZEP (400 nm) on the freshly cleaved surface of the HOPG. Then, remove the photoresist of the flake array area by electron beam lithography and use a brief oxygen plasma clean to remove the organic residues on the exposed HOPG surface. Third, deposit a 200 nm thick Pt film onto the array via electron beam evaporation and lift-off process. Finally, employ metal as a mask and employ reactive ion etching (oxygen ions) to obtain graphite flakes with Pt film, achieving an etching depth of 1 μm. Supplementary Figure S3 illustrates the characterizations of the graphite flakes.

### Atmosphere control

We utilized a custom-made 7-liter seamless aluminum chamber to make sure we maintained a stable local environment. Before conducting each experiment whether under dry nitrogen or humid nitrogen protection, the chamber underwent a 60-minute nitrogen gas flush at a flow rate of 2 liters per minute to ensure optimal conditions.

### Thermal annealing

Following the nitrogen flushing, the temperature was raised from room temperature to 150°C within 6 minutes using a heating device. Once stabilized at this temperature for 30 minutes, it gradually cooled back to room temperature over approximately 2 hours.

### Simulation protocols

The MD simulations in this work were carried out by the open-source code LAMMPS [54,55]. The intralayer interaction of graphene and Au were described by reactive empirical bond order potential (REBO) [56] and EAM potential [57], respectively; TIP4P/2005 model was used to describe the water molecules. The interlayer interaction between water and the Au (111) surface was tuned to correctly reproduce the experimental contact angle of 77°. Periodic boundary conditions were applied to the in-plane *x* and *y* directions. All simulations were performed under finite temperature *T* = 298 K, with a Nosé–Hoover thermostat applied to the Au substrate. The diffusion coefficient of the graphene slider was estimated from the Einstein relation. The structural factor was calculated by using [58,59]


\begin{eqnarray*}
S( {\boldsymbol{q}}) = \frac{1}{{{N}_O}}\mathop \sum \limits_{j = 1}^{{N}_O} \mathop \sum \limits_{i = 1}^{{N}_O} \exp \left[ {i{\boldsymbol{q}} \cdot \left( {{{\boldsymbol{r}}}_j - {{\boldsymbol{r}}}_i} \right)} \right]\end{eqnarray*}

where ${N}_O$ is the total number of oxygen atoms, and ${{\boldsymbol{r}}}_i$ represents the position of the *i*-th oxygen atom.

## Supplementary Material

nwae019_Supplemental_File
