# Supplementary material for: The anomalous effect of electric field on friction for microscale structural superlubric graphite/Au contact
Source: Natl Sci Rev. 2024 Jan 25;11(9):nwae019. doi: 10.1093/nsr/nwae019 (PMC11321252; doi:10.1093/nsr/nwae019)
Supplement: nwae019_Supplemental_File [file nwae019_supplemental_file.docx]

**Supplementary Materials**

**The anomalous effect of electric field on friction for microscale structural superlubric graphite/Au contact**

Yelingyi Wang^1,2^, Jin Wang^3,4^, Tielin Wu^1,2^, Weipeng Chen^1,2^, Deli Peng^5^, Zhanghui Wu^2*^, Ming Ma^1,5,6*^, Quanshui Zheng^1,2,5,7*^

^1^Center for Nano and Micro Mechanics, Tsinghua University, Beijing 100084, China.

^2^Department of Engineering Mechanics, School of Aerospace Engineering, Tsinghua University, Beijing 100084, China.

^3^International School for Advanced Studies (SISSA), Via Bonomea 265, Trieste, 34136, Italy.

^4^International Centre for Theoretical Physics (ICTP), Strada Costiera 11, Trieste, 34151, Italy.

^5^Institute of Superlubricity Technology, Research Institute of Tsinghua University in Shenzhen, Shenzhen 518057, China.

^6^Department of Mechanical Engineering, State Key Lab of Tribology in Advanced Equipment (SKLT), Tsinghua University, Beijing 10084, China.

^7^Tsinghua Shenzhen International Graduate School, Shenzhen 518057, China.

Corresponding authors:

wuzh1995@mail.tsinghua.edu.cn, maming16@tsinghua.edu.cn, zhengqs@tsinghua.edu.cn

This supplementary material contains the following contents:

1. The transfer process of graphite flake
2. Preparation of Au substrate and the height of Au pillar
3. Theoretical analysis of the full contact condition
4. The effect of cyclic voltage on the friction behavior of graphite/Au
5. The pick and flip process of graphite flake
6. The relationship of contact resistance and interface contact state
7. The effect of different direction of voltage on the friction behavior of graphite/Au
8. **The transfer process of graphite flake**

Firstly, we used a tungsten microtip to attach the Pt film of graphite flake, as shown in Fig. S1a, and applied a shear stress until the microtip and graphite flake split a short distance from their vertical direction, as shown in Fig. S1b. Secondly, we removed the microtip to observe whether the sheared graphite flake undergoes self-retraction motion (SRM)^1,2^ to determine whether it has a single crystal superlubric interface, as shown in Fig. S1c. Lastly, we re-attached the graphite flake which has SRM property with a microtip and split it out completely, as shown in Fig. S1d & S1e, after that, we removed the dangling graphite flake dragged by the microtip, and placed it slowly on the atomically smooth fabricated Au surface. Since the adsorption force of the graphite flake and Au is larger than that of the microtip and graphite flake, the graphite flake will remain on the Au surface^1-3^ (Fig. S1f).


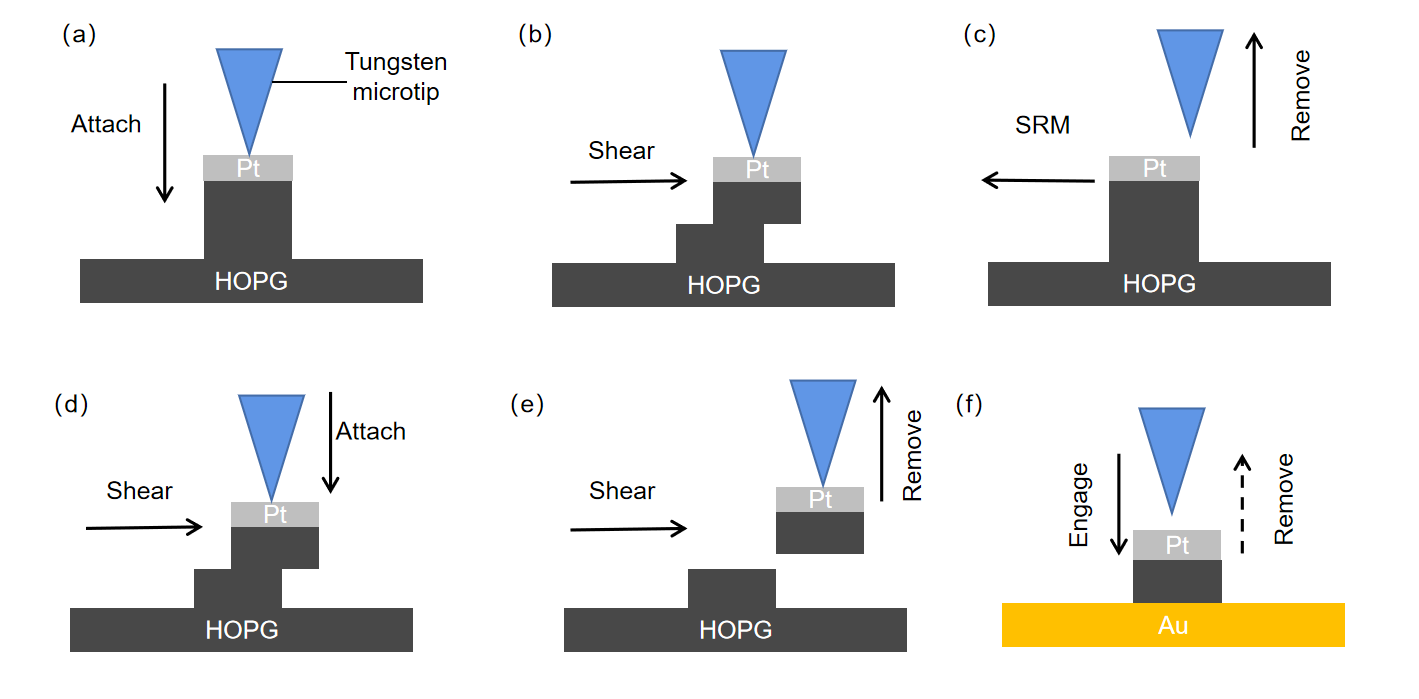
**Figure S1. The transfer process of graphite flake from pristine graphite mesa to Au substrate.** (a) Attachment of microtip and graphite flake with Pt film. (b,c) Observation of SRM. (d)-(f) Transfer process of graphite flake with SRM property.

1. **Preparation of Au substrate and the height map of Au pillar**

Figure S2 illustrates the step-by-step preparation process of the Au pillar, ranging in side lengths from 3 μm to 10 μm. Step 1: Spin-coat photoresist onto the silicon substrate. Step 2: Expose the photoresist and develop it to create an array of patterned photoresist. Step 3: Perform lithography on the silicon substrate to form an array of silicon pillars. Step 4: Remove the remaining photoresist and clean the substrate. Step 5: Utilize magnetron sputtering to deposit a 100 nm thick Au film onto the silicon substrate.


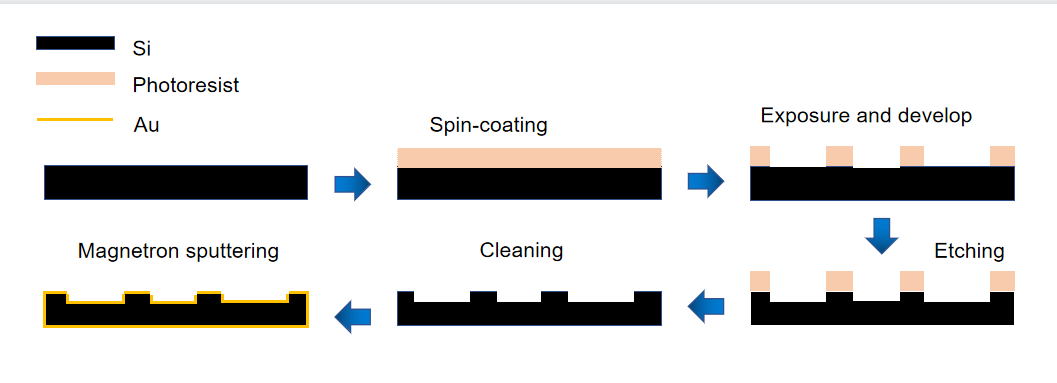


Step 5

Step 4

Step 3

Step1

Step 2

**Figure S2. The preparation process of the Au pillar.**

The thickness of the Au pillar was measured using tapping-mode AFM (Atomic Force Microscopy). In Fig. S3a, a representative height image of an Au pillar on the substrate is presented. The square pillar measures 6 μm in length, with a height of approximately 600 nm (refer to Fig. S3b). Notably, the Au pillars exhibit a flat surface without chamfers or burrs.


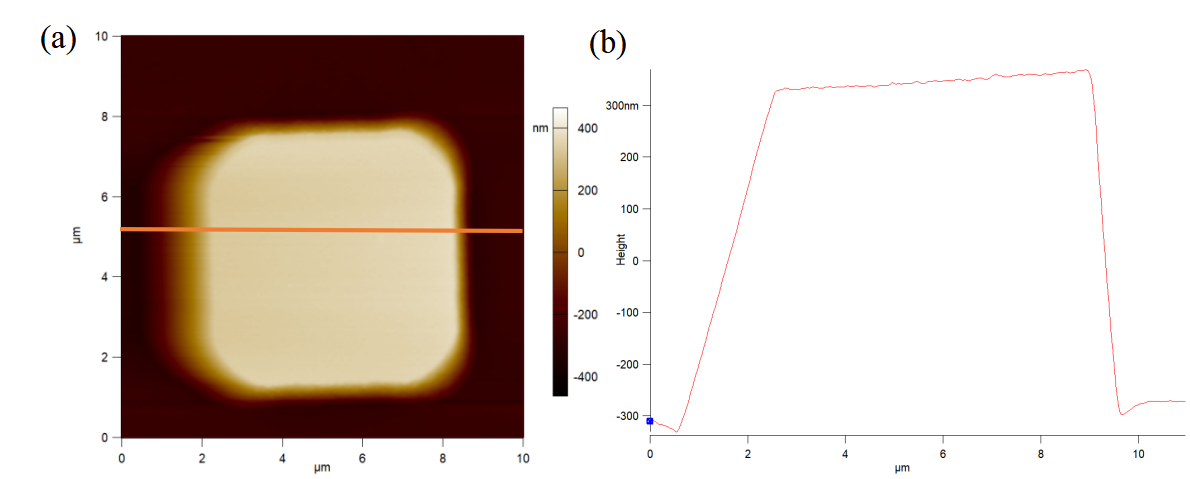


**Figure S3. The height map of an Au pillar on the substrate.** (a) Height map of a Au pillar. (b) The cross-sectional height map corresponding to the orange line of (a).

1. **Theoretical analysis of the contact condition**

According to the formular^4^ $H_{c}=3Eah^{2}/16\left( \gamma a+p_{\mathrm{zz}}ha \right)$, where *H*_c_ is the critical thickness of the graphite flake above which the graphite will fully contact with the substrate, *E* = 36.5 GPa is the Young’s modulus of graphite along the normal direction^5^, *a* = 4 μm is the side length of the graphite flake, *h* is the height of the rough peak, $\gamma=0.889 J/m^{2}$ is the surface energy^6^ and $p_{zz}=1 \mathrm{MPa}$ is the external normal stress, we can calculate the $H_{c}$ of our experimental system. We choose the rough peak with the largest curvature and height on the Au surfaces to consider its contact condition with the graphite, where *h*≈1.8 nm and *L*≈60 nm (Fig. 1d). We can finally obtain:

$$\begin{aligned} H_{c}=\frac{3Eah_{0}^{2}}{16\left( \gamma a+p_{zz}h_{0}a \right)}\approx19.5 \mathrm{nm}. \end{aligned}$$

Since the total thickness of graphite flake *H* in our experiments is around 300 nm in Fig. S4 and $p\mathrm{zz}$ is between 1 and 4 MPa, meaning $H\gg H_{c}$, it is reasonable to assume a full contact on the interface between graphite flake and the substrates.


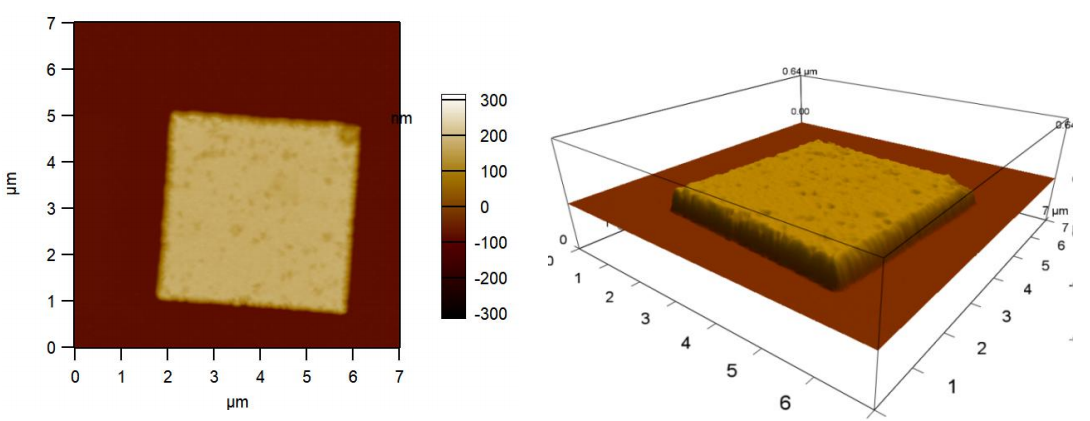


**Figure S4. The topography of the graphite flake by AFM scanning.**

1. **The effect of cyclic voltage on the friction behavior of graphite flake sliding on Au**

Figure S5 depicts the friction curves of a graphite flake sliding against Au, both in edge contact and in-plane contact, under the influence of different voltages (0.0 V, 1.0 V, 2.0 V, 3.0 V, and 4.0 V) applied repeatedly. Notably, a rapid response of the friction force was observed as the different voltages were alternately applied, causing the friction force to adjust to the corresponding values accordingly. Upon turning off the applied voltages, the friction force promptly returned to 1.3 μN (for edge contact) and 0.5 μN (for in-plane contact). This process was repeated, and once again, the friction force displayed a swift response and adapted to the respective values. These results indicate that the friction force of the graphite flake can be controlled in real-time and exhibits repeatability.


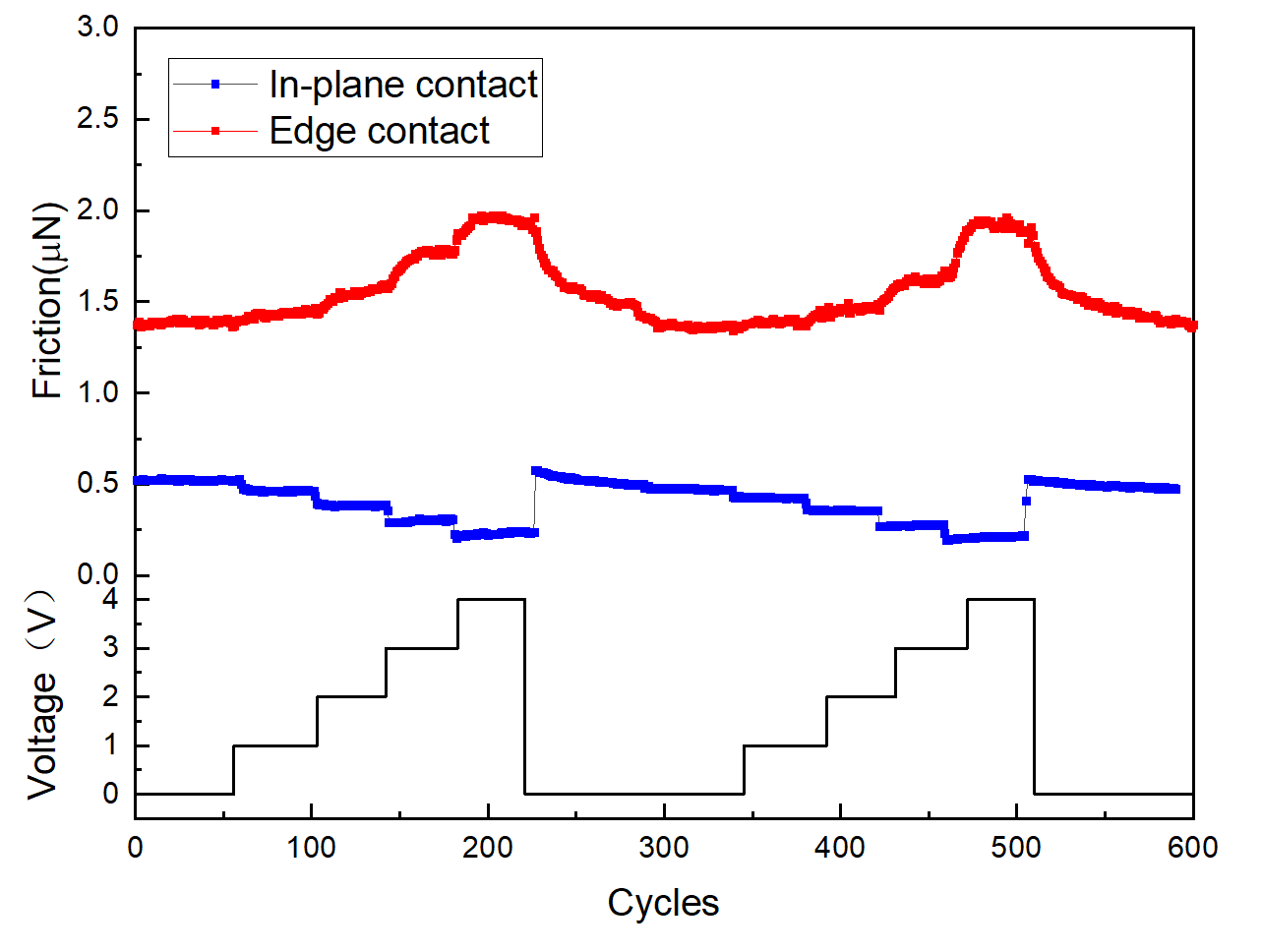


**Figure S5.** The effect of cyclic voltage on the friction behavior of graphite flake sliding on Au.

1. **The pick and flip process of graphite flake.**

As shown in Fig. S6, we use a tungsten microtip with a glue drop on the tip to attach the graphite flake and let the glue drop set a few minutes to dry. Since the adhesion between the flake and the Au surface is weaker than the adhesion between the glue and the Pt film of flake, the graphite flake can be picked up after the tip is removed. By turning the tip up, we can observe the lower surface of the graphite flake by Raman spectrum.


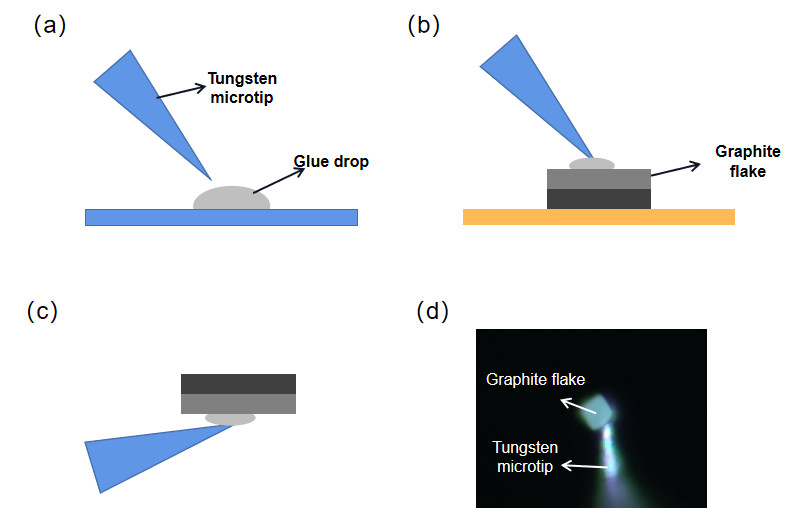


**Figure S6.** **The pick and flip process of graphite flake.** (a) Tungsten microtip attach on the glue; (b) Tungsten microtip attach on the flake and remove; (c) flip the tip to access the side of the graphite flake which points to the Au surface. (d) The light microscope image of the graphite flake.

1. **The variation of electrical resistance under dry and humid nitrogen environments.**

We conducted electrical resistance measurements on the graphite/Au interface under two different environmental conditions: dry nitrogen and water vapor environments, as shown in Fig. S7. To ensure continuous contact between the conductive probe and the graphite flake, we used an electronic balance during the experiments. Constant voltages were applied at the graphite/Au interface, and the electrical resistance of the system was initially measured around 50 Ω under the dry nitrogen environment.


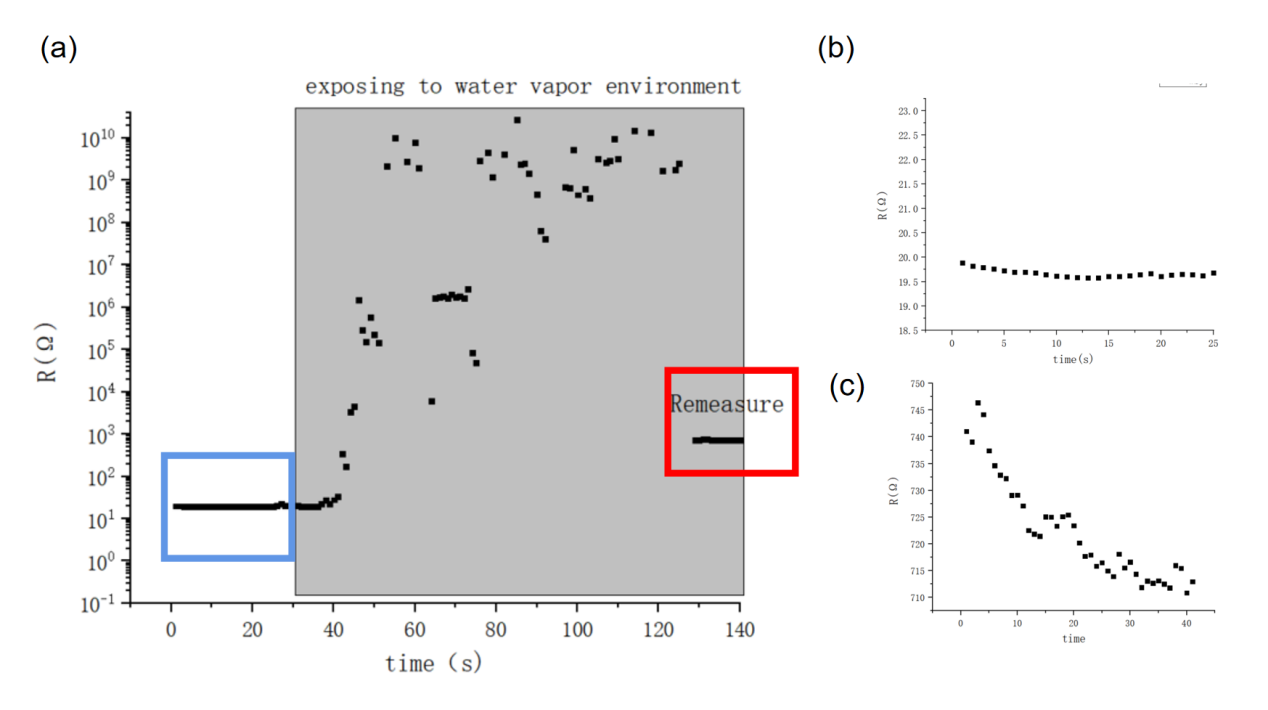


**[Figure S7](https://www.sciencedirect.com/science/article/pii/S0008622319310103#fig4). Electrical resistance of graphite/Au.** (a) Measurement of the electrical resistance of graphite/Au for in-plane contact under dry nitrogen environment and water vapor environment. (b) Electrical resistance in the blue box in (a). (c) Electrical resistance in the red box in (a).

Upon exposure to the water vapor environment, a significant change in electrical resistance occurred, reaching the order of ${10}^{10}$ Ω. After subjecting the system to several hundred friction loops, we observed the electrical resistance returning to 700 Ω. This fluctuation in electrical resistance suggests variations in the contact state between the graphite and Au surfaces, accompanied by the reappearance of water contaminants at the interface. These results indicate that water molecules can influence the electrical resistance at the graphite/Au interface, thus affecting the contact state between the materials.

1. **The effect of different direction of voltage on the friction behavior of graphite/Au**

We have changed the direction of the voltage, as shown in Fig. S8. It can be observed that no matter the electric field in which direction, the friction force for edge contact shows a trend of increase, and the samples for in-plane contact show a trend of decrease. There is a slight discrepancy in the specific values of friction force due to the different directions in which the voltage is applied. It should be the different orientation of water molecules that causing the difference of friction.


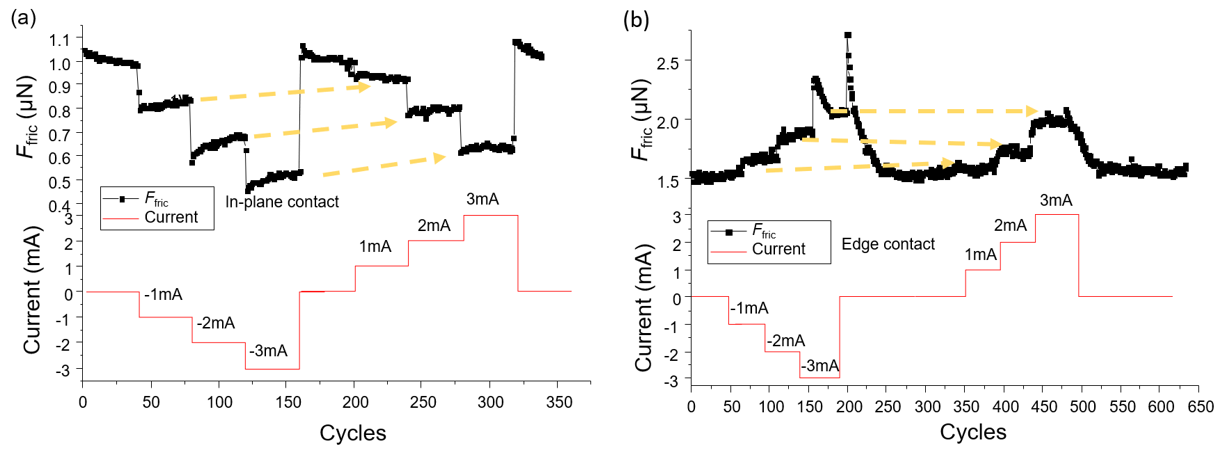


**Figure S8.** **The variation of friction with different direction of voltages.** (a) for in-plane contact; (b) for edge contact.

Concerning the direction dependence, we perform additional simulations to estimate the friction with parameter *E*= −1 V/nm. Results shows that the diffusion coefficient for this inversed electric field case is *D*=1.82±0.1 Å^2^/ps, which is 17% larger than *E*=1 V/nm case and 37% larger than the *E*=0 case. The result indicates that the friction-weakening behavior depends weakly on the direction of electric field, which again, agrees with our experimental observations.

This weakly direction-dependence can be understood by the orientation of the intercalated water molecule. Statistically, when the electric field is positive (pointing down, from graphite slider to Au substrate), the oxygen in the water molecules is closer to the graphite surface and has a relatively stronger interaction; when the electric field is negative (pointing up, from Au substrate to graphite slider), it is hydrogen that close to graphite and the interaction is weaker. The above understanding is based on the fact that the Au surface is hydrophilic and the graphite surface is hydrophobic, and the sliding interface is between water layer and graphite.

**Reference**

1 Hod, O., Meyer, E., Zheng, Q. & Urbakh, M. Structural superlubricity and ultralow friction across the length scales. *Nature* **563**, 485-492 (2018). <https://doi.org:10.1038/s41586-018-0704-z>

2 Liu, Z. *et al.* Observation of microscale superlubricity in graphite. *Phys Rev Lett* **108**, 205503 (2012). <https://doi.org:10.1103/PhysRevLett.108.205503>

3 Song, Y. *et al.* Robust microscale superlubricity in graphite/hexagonal boron nitride layered heterojunctions. *Nature Materials* **17**, 894-899 (2018). <https://doi.org:10.1038/s41563-018-0144-z>

4 Peng, D. *et al.* 100 km wear-free sliding achieved by microscale superlubric graphite/DLC heterojunctions under ambient conditions. *Natl Sci Rev* **9**, nwab109 (2022). <https://doi.org:10.1093/nsr/nwab109>

5 Liu, Z. *et al.* Interlayer binding energy of graphite: A mesoscopic determination from deformation. *Physical Review B* **85**, 205418 (2012). <https://doi.org:10.1103/PhysRevB.85.205418>

6 Heyraud, J. C. & Metois, J. J. Equilibrium shape of gold crystallites on a graphite cleavage surface: Surface energies and interfacial energy. *Acta Metallurgica* **28**, 1789-1797 (1980). <https://doi.org:https://doi.org/10.1016/0001-6160(80)90032-2>
